# Supplementary material for: Ca2+-activated Cl− channel TMEM16A/ANO1 identified in zebrafish skeletal muscle is crucial for action potential acceleration
Source: Nat Commun. 2019 Jan 10;10:115. doi: 10.1038/s41467-018-07918-z (PMC6328546; doi:10.1038/s41467-018-07918-z)
Supplement: Supplementary file 1 — Supplementary Information [file 41467_2018_7918_MOESM1_ESM.pdf]

**Supplementary Information**

# $Ca^{2+}$-activated $Cl^-$ channel TMEM16A/ANO1 identified in zebrafish skeletal muscle is crucial for action potential acceleration

**Dayal et al.**

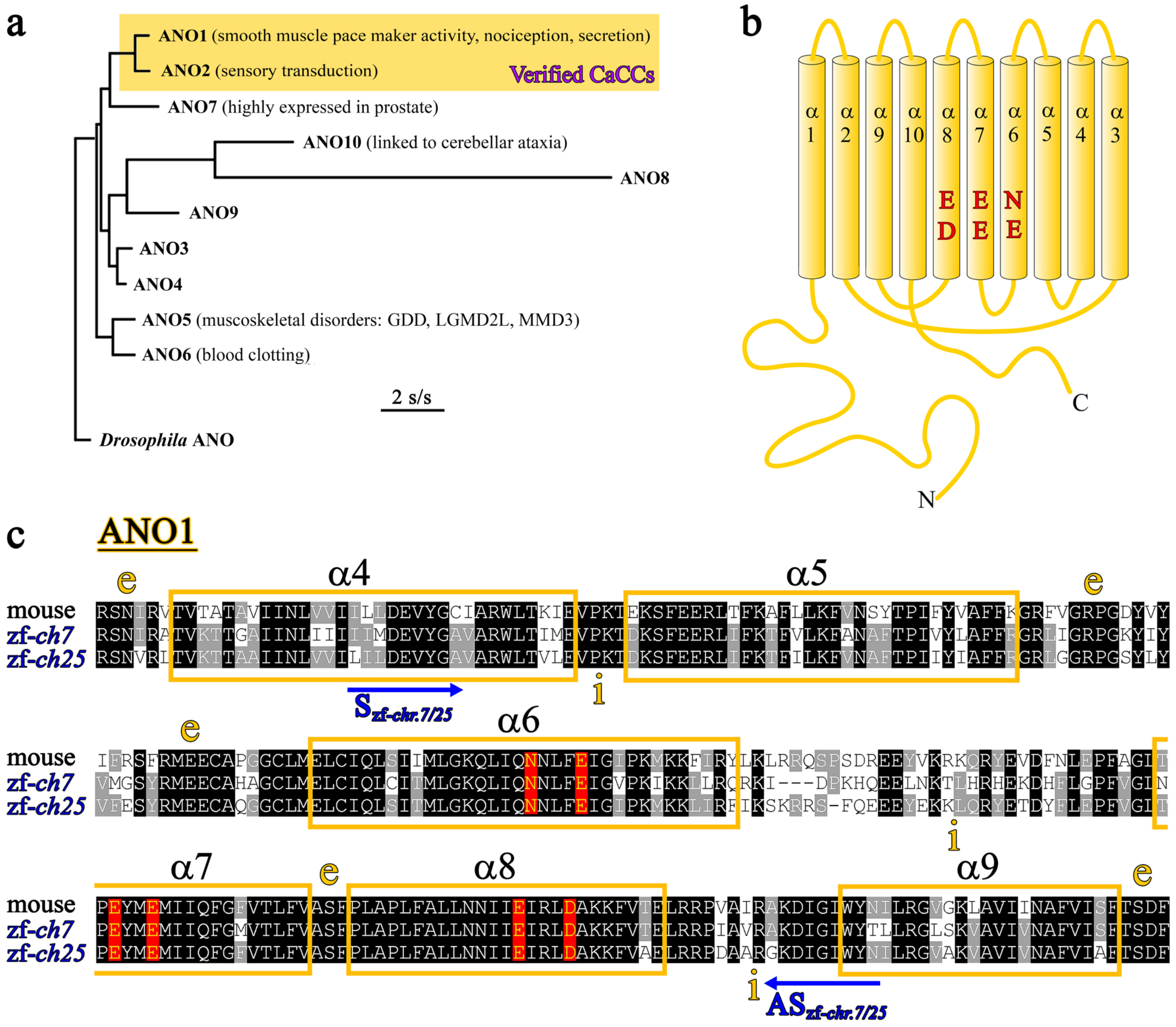


**Supplementary Figure 1. Selection and design of RT-PCR primers for the two verified CaCCs, ANO1 and ANO2**

**a** Likelihood-based phylogenetic tree (Treefinder, version 2011) from aligned amino acid sequences of all isoforms of the mouse ANO family with the ancestral *Drosophila* ANO channel (GenBank accession no. NP_648535) as outgroup. Yellow box embraces the only two verified CaCCs, ANO1 (accession no. NP_848757) and ANO2 (accession no. NP_705817). Rest of the ANO isoforms namely, ANO3 (NP_001121575), ANO4 (NP_001264117), ANO5 (NP_808362), ANO6 (NP_001240742), ANO7 (NP_996914), ANO8 (XP_017168360), ANO9 (XP_006536311), and ANO10 (NP_598740) have either unknown functions or known physiological and pathological impacts as indicated. Recessive ANO5 mutations are linked to: GDD, gnathodiaphyseal dysplasia[1, 2]; LGMD2L, limb-girdle muscular dystrophy type 2L[3], and MMD3, Miyoshi muscular dystrophy-3[3, 4]. Likelihood score, -22,527.7; edge lengths optimized. Scale bar, evolutionary distance in substitutions per site (s/s). **b** Proposed membrane topology of ANO channels with 10 transmembrane helices α1 – α10[5]. The 6 residues N650, E654, E702, E705, E734, and D738, assumed to form the ANO $Ca^{2+}$-binding site[5, 6] are indicated in red. N- and C-terminus are at the intracellular side. **c** Sequence alignment of mouse ANO1 protein fragment from α4 to α9 with corresponding zebrafish ANO1-a isoform encoded from chromosome 7 (zf-*ch7*) and ANO1-b isoform from chromosome 25 (zf-*ch25*). Transmembrane helices (α4 to α9) are boxed in yellow and residues forming the putative $Ca^{2+}$-binding site in red. *e* indicates the extracellular side and *i* the intracellular side of the transmembrane core. Positions of sense and antisense primer for both ANO1-a and ANO1-b isoforms ($S_{zf\text{-}chr.7/25}$ and $AS_{zf\text{-}chr.7/25}$) are indicated with blue arrows. Accordingly at corresponding positions, ANO2-a and ANO2-b specific primers were designed. The sequences of all primers are specified in Supplementary Table 1.

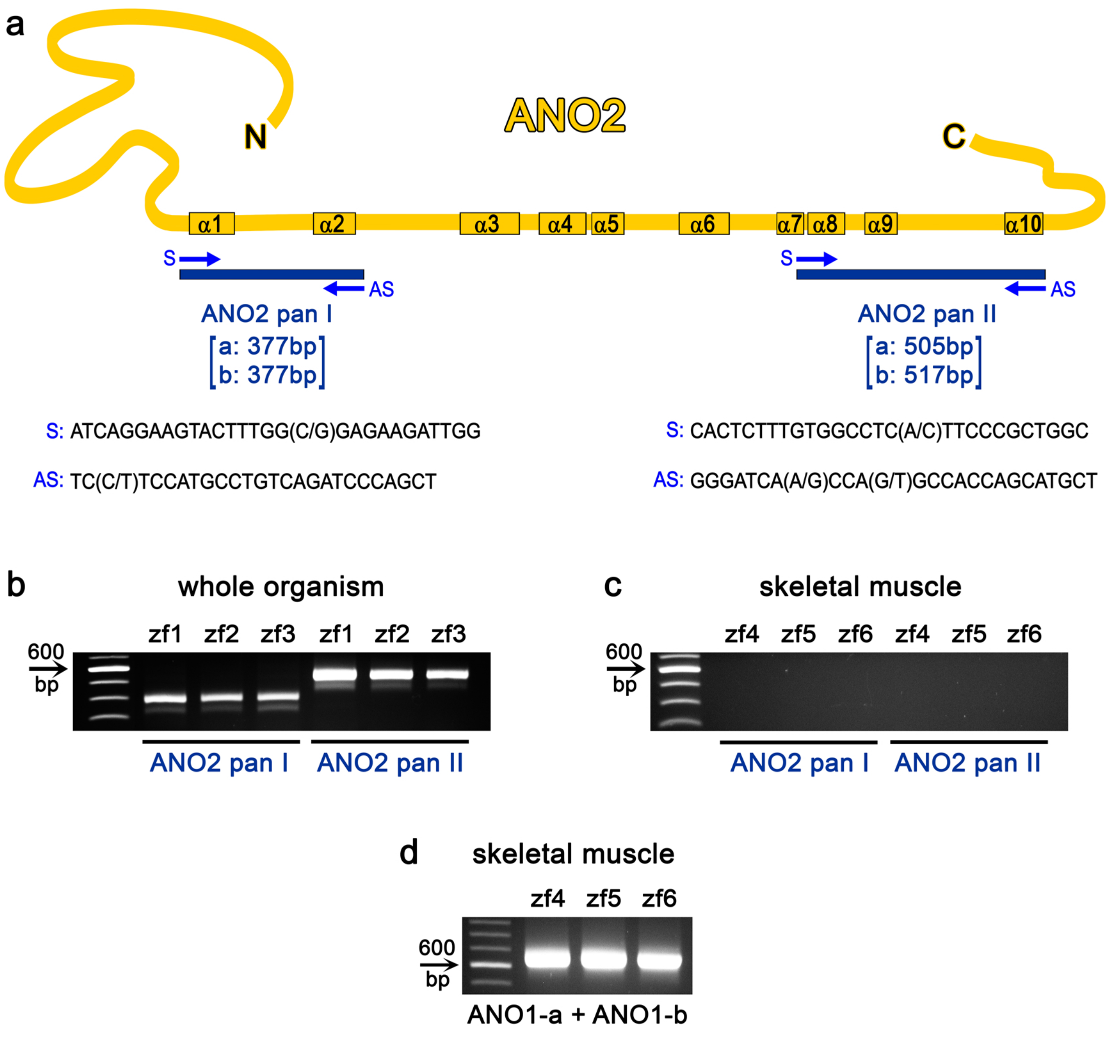


**Supplementary Figure 2. Design and position of two RT-PCR pan-primer pairs to confirm the absence of ANO2 isoforms in skeletal muscle**

**a** Schematic linear representation of ANO2 isoforms showing the 10 transmembrane helices α1 – α10[5] (yellow boxes). Blue arrows indicate the positions of the degenerate sense (S) and antisense (AS) ANO2 pan-primer pairs and blue bars the expected fragment sizes (in bp), generated from ANO2-a (a) and ANO2-b (b). The sequences of the two ANO2 pan-primer pairs are depicted below the respective bars. **b** RT-PCR amplification from first strands of 3 adult normal whole zebrafish (zf1 - zf3) showed robust amplification products with both ANO2 pan-primer pairs. **c** In contrast, RT-PCR amplification with the same ANO2 pan-primer pairs and under identical PCR conditions did not show any signals from first strands specifically derived from skeletal-muscle of 3 adult normal zebrafish (zf4 - zf6). **d** However, control PCR amplication with a stochiometric mix of ANO1-a and ANO1-b primers (Supplementary Table 1) with the same first strands derived from skeletal muscles of zebrafish zf4 - zf6 produced strong signals.

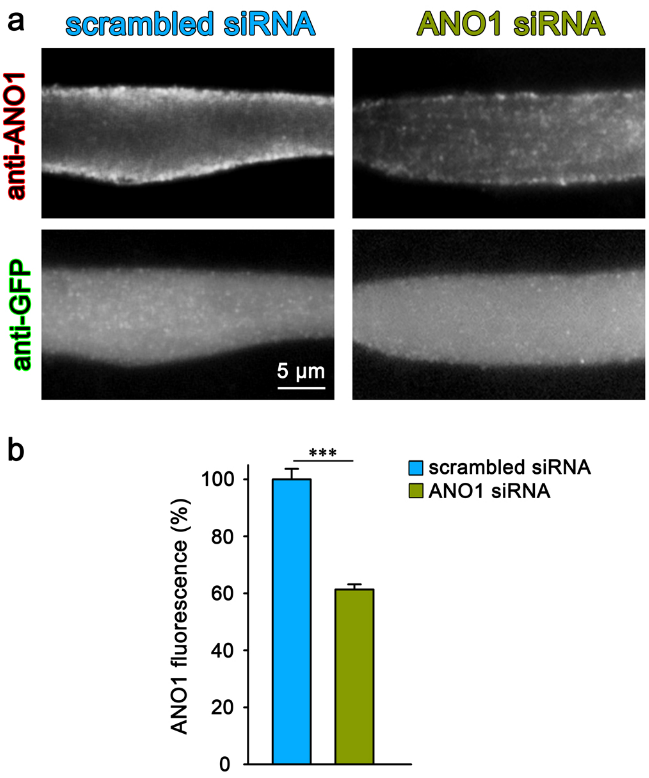


**Supplementary Figure 3. siRNA knock down of ANO1 confirms its sarcolemmal localisation**
Representative images of normal zebrafish myotubes double immunolabelled for ANO1 (anti-ANO1) and the CFP-tag of shRNA constructs (anti-GFP). **a** The ANO1 sarcolemmal expression (upper left image) is unaltered by control scrambled siRNA expression (GFP signal, lower left image). In contrast, surface membrane expression of ANO1 is considerably reduced in myotubes (upper right image) expressing siRNA 2073-2093 (ANO1-b numbering) (GFP signal, lower right image) targeted against both ANO1-a/b isoforms. **b** Quantification of fluorescence intensity of ANO1 (see Methods) exhibited a significant ($P <$ 0.001) reduction in the ANO1 surface membrane signal upon expression of siRNA 2073-2093 (61.4 ± 1.8%, $n$ = 128) compared to control scrambled siRNA-expressing myotubes (100.0 ± 3.7%, $n$ = 161). Data are presented as mean ± s.e.m.; *** $P < 0.001$ determined by unpaired Student's *t*-test.

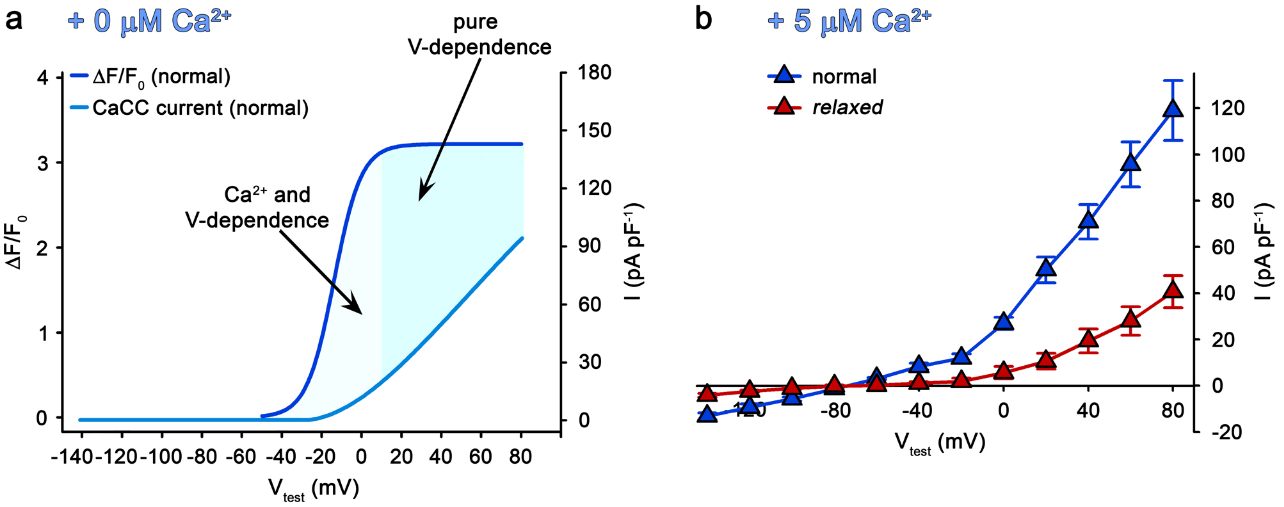


**Supplementary Figure 4. ANO1 is gated synergistically by membrane potential and intracellular $Ca^{2+}$ concentration**

**a** Overlay of the CaCC I-V curve (curve-fit from Fig. 6b) and the corresponding intracellular $Ca^{2+}$ release curve (curve-fit from Fig. 1d) obtained from normal zebrafish myotubes at physiological conditions (+ 0 µM $Ca^{2+}$). Light blue indicates the zone of combined $Ca^{2+}$- and voltage dependence of the ANO1 current and darker blue, the zone of pure voltage dependence due to the saturation with $Ca^{2+}$ ions by SR release. **b** Outward rectification of ANO1 currents after cytosolic addition of low (+ 5 µM) $Ca^{2+}$ via the patch pipette solution. ANO1 currents were recorded with a 3-s pulse protocol from normal ($n$ = 9) and *relaxed* myotubes ($n$ = 6). Data are presented as mean ± s.e.m.

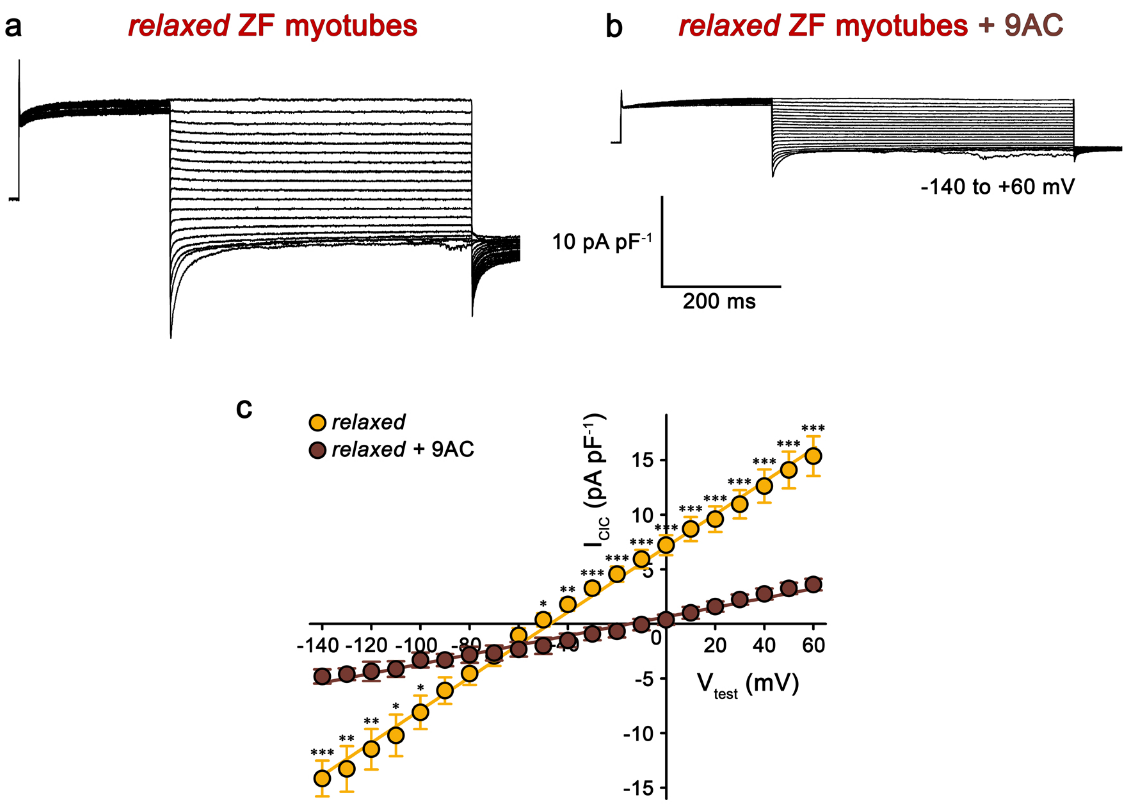


**Supplementary Figure 5. 9AC block of ClC channels confirms the existence of ClC currents in zebrafish skeletal muscle**

**a, b** Representative ClC current traces from *relaxed* myotubes elicited using the pulse protocol described in Fig. 7a in the (**a**) absence and (**b**) presence of 1 mM 9AC, a ClC channel blocker[7]. Scale bars, 200 ms (horizontal), 10 pA $pF^{-1}$ (vertical). **c** Voltage dependence of ClC currents recorded from *relaxed* myotubes display significant ($P < 0.05$ – $P < 0.001$) difference between ClC currents obtained in the absence ($n$ = 10) and presence ($n$ = 9) of 9AC, with I = 15.37 ± 1.81 pA $pF^{-1}$ and 3.61 ± 0.53 pA $pF^{-1}$, respectively, at +60 mV and I = -14.15 ± 1.64 pA $pF^{-1}$ and -4.81 ± 0.65 pA $pF^{-1}$, respectively, at -140 mV. Data are presented as mean ± s.e.m.; * $P < 0.05$, ** $P < 0.01$, *** $P < 0.001$ determined by unpaired Student's *t*-test.

**Supplementary Table 1**

**Sequences of PCR primers used for analysing the expression profiles of ANO1 and ANO2 isoforms in superficial slow/red and deep fast/white skeletal muscles of zebrafish.**

| **Target Gene and its location** | **Forward primer (5’ - 3’)** | **Reverse primer (5’ - 3’)** | **PCR fragment size** |
|---|---|---|---|
| **ANO1-a Chromosome 7** | ATCATCATGGATGAGG TCTACGGAGCAG | AGTGTACCAGATTCCG ATGTCCTTTGCTC | 627 bp |
| **ANO1-b Chromosome 25** | CTGATCCTGGATGAAG TTTACGGAGCCG | ATTATACCAGATTCCG ATGTCTTTGCCTC | 633 bp |
| | | | |
| **ANO2-a Chromosome 4** | ATTTTGGATGAAATCT ATGGCATGGTGG | GTTATACCAGATGCCA ATGTCTTTGGTCC | 633 bp |
| **ANO2-b Chromosome 18** | GTGCTGGATGAGATCT ACGGTGCAATAG | GTTGTACCATATGCCA ATCTCTTTAGCAC | 639 bp |
| | | | |
| **ANO2-a/b Chromosome 4/18** | ATCAGGAAGTACTTTG G(C/G)GAGAAGATTGG | TC(C/T)TCCATGCCTGT CAGATCCCAGCT | 377/377 bp |
| **ANO2-a/b Chromosome 4/18** | CACTCTTTGTGGCCTC (A/C)TTCCCGCTGGC | GGGATCA(A/G)CCA(G/ T)GCCACCAGCATGCT | 505/517 bp |
| | | | |
| **DHPR $\alpha_{1S}$-a** | ACCAATAAGGCGCTGT TGTCTTCTG | TTCGTCTCACGCGGTC ATCTGGAA | 385 bp |
| **DHPR $\alpha_{1S}$-b** | GATGTATGCATTCGGT CCGAGAAG | GATTCCTGGAGATTTG GGTCCA | 460 bp |

**Supplementary Table 2**

**Composition of patch pipette solutions with different free $Ca^{2+}$ concentrations.**

| **Free [$Ca^{2+}$ (µM)** | **0** | **2** | **5** | **7.5** | **13** |
|---|---|---|---|---|---|
| **Cs-aspartate (mM)** | 100 | 100 | 100 | 100 | 100 |
| **$CsCl_2$ (mM)** | 45 | 45 | 45 | 45 | 45 |
| **$MgCl_2$ (mM)** | 2 | 2 | 2 | 2 | 2 |
| **Mg-ATP (mM)** | 2 | 2 | 2 | 2 | 2 |
| **HEPES (mM)** | 10 | 10 | 10 | 10 | 10 |
| **Cs-EGTA (mM)** | 0.1 | 0.1 | 1 | 1 | 2.5 |
| **$CaCl_2$ (mM)** | 0 | 0.1 | 0.5 | 1 | 2.5 |

Free $Ca^{2+}$ levels in patch pipette solutions were achieved by calibrating the concentrations of Cs-EGTA and $CaCl_2$ (boxed in gray), calculated with the MaxChelator simulation program (http://maxchelator.stanford.edu).

## Supplementary References


1. Marconi, C. et al. A novel missense mutation in ANO5/TMEM16E is causative for gnathodiaphyseal dyplasia in a large Italian pedigree. *Eur. J. Hum. Genet.* **21(6),** 613–619 (2013).

2. Tsutsumi, S. et al. The Novel Gene Encoding a Putative Transmembrane Protein Is Mutated in Gnathodiaphyseal Dysplasia (GDD). *Am. J. Hum. Genet.* **74(6),** 1255-1261 (2004).

3. Bolduc, V. et al. Recessive mutations in the putative $Ca^{2+}$-activated $Cl^{-}$ channel Anoctamin 5 cause proximal LGMD2L and distal MMD3 muscular dystrophies. *Am. J. Hum. Genet*. **86(2),** 213-221 (2010).

4. Linssen, W. H. et al. Genetic heterogeneity in Miyoshi-type distal muscular dystrophy. *Neuromuscul. Disord.* **8(5),** 317-320 (1998).

5. Brunner, J. D., Lim, N. K., Schenck, S., Duerst, A. & Dutzler, R. X-ray structure of a calcium-activated TMEM16 lipid scramblase. *Nature* **516,** 207-212 (2014).

6. Pedemonte, N. & Galietta, L. J. Structure and function of TMEM16 proteins (anoctamins). *Physiol. Rev.* **94(2),** 419-459 (2014).

7. Lueck, J. D., Rossi, A. E., Thornton, C. A., Campbell, K. P. & Dirksen, R. T. Sarcolemmal-restricted localization of functional ClC-1 channels in mouse skeletal muscle. *J. Gen. Physiol.* **136(6),** 597-613 (2010).
