# Supplementary material for: Ca2+-activated Cl− channel TMEM16A/ANO1 identified in zebrafish skeletal muscle is crucial for action potential acceleration
Source: Nat Commun. 2019 Jan 10;10:115. doi: 10.1038/s41467-018-07918-z (PMC6328546; doi:10.1038/s41467-018-07918-z)
Supplement: Supplementary file 3 — Reporting Summary [file 41467_2018_7918_MOESM3_ESM.pdf]

nature research

Corresponding author(s): Dr. Manfred Grabner
Dr. Anamika Dayal

Reporting Summary

Nature Research wishes to improve the reproducibility of the work that we publish. This form provides structure for consistency and transparency in reporting. For further information on Nature Research policies, see Authors & Referees and the Editorial Policy Checklist.

Statistical parameters

When statistical analyses are reported, confirm that the following items are present in the relevant location (e.g. figure legend, table legend, main text, or Methods section).

n/a	Confirmed	
☐	☒	The exact sample size (*n*) for each experimental group/condition, given as a discrete number and unit of measurement
☐	☒	An indication of whether measurements were taken from distinct samples or whether the same sample was measured repeatedly
☐	☒	The statistical test(s) used AND whether they are one- or two-sided<br>*Only common tests should be described solely by name; describe more complex techniques in the Methods section.*
☒	☐	A description of all covariates tested
☒	☐	A description of any assumptions or corrections, such as tests of normality and adjustment for multiple comparisons
☐	☒	A full description of the statistics including central tendency (e.g. means) or other basic estimates (e.g. regression coefficient) AND variation (e.g. standard deviation) or associated estimates of uncertainty (e.g. confidence intervals)
☐	☒	For null hypothesis testing, the test statistic (e.g. *F*, *t*, *r*) with confidence intervals, effect sizes, degrees of freedom and *P* value noted<br>*Give P values as exact values whenever suitable.*
☒	☐	For Bayesian analysis, information on the choice of priors and Markov chain Monte Carlo settings
☒	☐	For hierarchical and complex designs, identification of the appropriate level for tests and full reporting of outcomes
☒	☐	Estimates of effect sizes (e.g. Cohen's *d*, Pearson's *r*), indicating how they were calculated
☐	☒	Clearly defined error bars<br>*State explicitly what error bars represent (e.g. SD, SE, CI)*

Our web collection on statistics for biologists may be useful.

Software and code

Policy information about availability of computer code

Data collection	MetaVue image processing software (v 6.2); PCLAMP (v 7.0); Patchmaster (v 2.72).
Data analysis	ImageJ (open source); BLOCK-iT™ RNAi Designer software; MetaVue image processing software (v 6.2); MaxChelator simulation program (http://maxchelator.stanford.edu); ClampFit (v10.0); SigmaPlot (v 10.0); FitMaster (v 2x73.2); Treefinder (v 2011).

For manuscripts utilizing custom algorithms or software that are central to the research but not yet described in published literature, software must be made available to editors/reviewers upon request. We strongly encourage code deposition in a community repository (e.g. GitHub). See the Nature Research guidelines for submitting code & software for further information.

Data

Policy information about availability of data

All manuscripts must include a data availability statement. This statement should provide the following information, where applicable:

- Accession codes, unique identifiers, or web links for publicly available datasets
- A list of figures that have associated raw data
- A description of any restrictions on data availability

Data supporting the findings of this study are available from the corresponding authors upon reasonable request

Field-specific reporting

Please select the best fit for your research. If you are not sure, read the appropriate sections before making your selection.

☒ Life sciences ☐ Behavioural & social sciences ☐ Ecological, evolutionary & environmental sciences

For a reference copy of the document with all sections, see nature.com/authors/policies/ReportingSummary-flat.pdf

Life sciences study design

All studies must disclose on these points even when the disclosure is negative.

Sample size	Sample sizes of zebrafish myotubes or tissues are based on previous publications, hence, power calculations were not necessary.
Data exclusions	In our study, no data points or samples were excluded from analyses.
Replication	All replication attempts were successful.
Randomization	For siRNA transfection or drug administration assays, myotube cultures according to genotype were arbitrarily allocated to the specific sample groups without the use of an explicit randomization procedure.
Blinding	Experiments did not require blinding and thus, were not performed under blinded conditions.

Reporting for specific materials, systems and methods

Materials & experimental systems

n/a	Involved in the study
☒	☐ Unique biological materials
☐	☒ Antibodies
☒	☐ Eukaryotic cell lines
☒	☐ Palaeontology
☐	☒ Animals and other organisms
☒	☐ Human research participants

Methods

n/a	Involved in the study
☒	☐ ChIP-seq
☒	☐ Flow cytometry
☒	☐ MRI-based neuroimaging

Antibodies

Antibodies used

Primary antibodies:
1) mouse monoclonal antibody 1A against DHPRα1S (Affinity Bioreagents, MA3-920)
2) rabbit anti-GFP (Invitrogen, A11122)
3) rabbit polyclonal anti-ANO1 (Abcam, ab84115)
4) goat polyclonal anti-ANO5 (Santa Cruz, sc-169628)

Secondary antibodies:
1) Goat anti-mouse Alexa Fluor 594 (Invitrogen, A11032)
2) Goat anti-rabbit Alexa Fluor 488 (Invitrogen, A11034)
3) Rabbit anti-goat Cy3 (Sigma, C2821)
4) Rabbit anti-mouse FITC (Sigma, F9137)

Validation

All antibodies were stated to be applicable for immunofluorescence experiments and were validated based on the validation information provided by the manufacturer. These antibodies have been used in numerous studies cited on the manufacturers' websites. All the antibodies successfully labeled the expected targets (DHPRα1S, Ano1, Ano5 and GFP tag) and the images are included in the study.

Animals and other organisms

Policy information about studies involving animals; ARRIVE guidelines recommended for reporting animal research

Laboratory animals	zebrafish strains were obtained from Max Planck Institute Tübingen, Germany; No sex differentiation was needed.

Wild animals	n/a
Field-collected samples	n/a
